# Editorial

**Published:** 1913-10

**Authors:** 


### EDITORIAL

The Business Office of the *Journal-Record* is 23 West Alabama Street.

The Editorial Office is 1014-15 Atlanta National Bank Building.

Address all Business Communications to *Journal-Record of Medicine*, 23 West Alabama Street.

Make remittances payable to *The Journal-Record of Medicine.*

On matters pertaining to the Editorial and Original Communications, address **Edgar G. Ballenger, M. D., Atlanta, Ga.**

Reprints of Original Articles will be furnished at cost price. **Requests for the same** should always be made in THE MANUSCRIPT.

We will present, postpaid, on request to each contributor of **an original article,** twenty (20) marked copies of *The Journal-Record of Medicine* containing **such article.**

### DRUG SUBSTITUTION—THE REMEDY.

Our attention is called to another "substitution" alleged to be practiced extensively by the druggists upon the doctors almost before their eyes and at which the perpetrators are said to be laughing because it goes unnoticed. We find it difficult to believe that anyone is laughing at it, for no one who knows enough to substitute drugs can think that such action is a laughing affair, no matter how much one may profit by it. It is against the law and when proved to have occurred it subjects one to punishment. This fact will appeal to the substituting mind even if the danger to suffering patients will not. But in either event, it is serious and demands our earnest attention.

The particular offense before us is the sale of packages of neo-salvarsan containing .6 gm. instead of .9 gm. which is the established dose, at the price of the full dose, .9 gm. It appears that there is a label on the package saying that the dose is smaller and therefore that the seller can say he is not defrauding on that account. But this serves only to make the matter more of a betrayal of confidence because the physician has a right to expect that the druggist would tell him of the difference if he knew it. The failure to do so lends a quality of meanness to the transaction that one dislikes to ascribe any man with whom he has business dealings.

We are told that these small packages which mask as the complete ones, are really smuggled into the country and are unlawful in every way. They offer a temptation to the impecunious salesman because they can be sold at the same price as the regular full package and they cost him considerably less. As to whether their contents are real neo-salvarsan or not, we are not prepared to say, but the firm that will attempt to deceive as to dosage will not hesitate to steal as to quality.

The dangers of uncertain and unknown dosing are too well known to describe, but when so much is made to depend upon the necessary doses as there is in these injections, the dangers are obviously greater than usual.

As to the remedy for this and similar conditions we have this to say:

Doubtless every druggist in business for a year has at least one doctor who is his personal friend, often stopping in the store for a social chat and establishing the cordial relations that grow up between men engaged in allied occupations or professions. Every such doctor would say that his friend the druggist would not substitute, no matter what others might do. Thus the whole field is covered and we seem to have shown that there is no substitution. The corollary is that no druggist will want his friend the doctor to know he substitutes and he will go a long way to prevent that knowledge getting out if he is guilty. The doctor will not tolerate substitution if for no other reason than that he knows he will get bad results. Thus if the doctor will take a live interest in the drugs he is using and let his friend the druggist understand in every instance that nothing but righteous drugs are to be used in their mutual affairs, there will be none but good drugs in stock. We are appealing to the "get together" spirit and we believe that more can be accomplished through it than by any other means.

The Fulton County Medical Society of Atlanta has instructed its Secretary to notify all its members as to the various substitutions in the present market and to warn them against the danger. The doctor's friendly interest with the druggist may well do more than appeals to the laws to free us from these conditions.

The doctors determine the amount in each package before administering the remedy.

R. R. Daly, M. D.

